# Water Supply in Relation to Health and Disease
*Snow (John, M.D.). On the Mode of Communication of Cholera. London: 1855.Snow (John, M.D.). Continuous Molecular Changes. London: 1853.Johnston (Professor J. F. W.). On the Water recently used in the Durham Jail. Monthly Journal of Medicine: May 1855.Hassall (A. H., M.D.). Microscopic Examination of London Water. London: 1850.Ainsworth (William). Observations on the Pestilential Cholera (Cholera Asphyxia) as it appeared in Sunderland in the Months of November and December 1831.Papers respecting the Origin, &c., of Yellow Fever in Bermuda in 1853. London: 1855.Budd (William, M.D.). Cholera: its Cause and Prevention. Association Medical Journal: 1854 and 1855.Carpenter (Alfred). Impure Water a Cause of Disease. Association Medical Journal, October 6, 1854.Lindsay (W. Lauder, M.D.). Experiments on the Communicability of Cholera to the Lower Animals. Edinburgh Med. and Surg. Journal: 1854.Mitchell (John). On the Falsifications of Foods : Article—Water and its Impurities.Letter from the President of the General Board of Health to Viscount Palmerston, accompanying a Report from Dr. Sutherland on Epidemic Cholera in the Metropolis in 1854. Blue Book: 1855.Report of the General Board of Health of the supply of Water to the Metropolis. Blue Book: 1850.Blackburne (William, M.D.). Scarlet Fever, and the Origin of Acute Contagions. London: 1803.The Registrar-General's Weekly Returns for 1854: England.Shapter (Thomas, M.D.). Sanitary Measures and their Results. London and Exeter: 1853.Minutes of Information collected with reference to Works for the Removal of Soil, Water, or Drainage of Dwelling Houses and Public Edifices. Published by the Board of Heath: 1852.Becker (F. W., M.D., F.R.S.). Letters on Cholera in Prussia in 1831. London: 1832.


**Published:** 1855-06

**Authors:** B. W. Richardson


### REVIEWS.

### (CRITICAL, HISTORICAL, AND ANALYTICAL.)

---

### WATER SUPPLY IN RELATION TO HEALTH AND DISEASE.*

THE question of water supply in its bearings on health is one which has, at various periods in the world's history, attracted the attention of those whose minds have been directed to the investigation of the nature and causes of disease: even Hippocrates referred to the effects of impure water in producing various disorders. The subject, however, has within these last four or five years received a degree of attention quite unprecedented, in consequence of the fact that a London physician, whose scientific reputation in the medical world stands deservedly high, has, in a manner neither to be overlooked nor forgotten, made the bold statement that the last two epidemics of cholera were mainly due to the distribution of impure water; and that to this cause alone *all* epidemics of cholera are chiefly attributable. But, not content with making this broad and remarkable assertion, Dr. Snow, the author to

---

* SNOW (John, M.D.). On the Mode of Communication of Cholera. London: 1855.

SNOW (John, M.D.). Continuous Molecular Changes. London: 1853.

JOHNSTON (Professor J. F. W.). On the Water recently used in the Durham Jail. Monthly Journal of Medicine: May 1855.

HASSALL (A. H., M.D.). Microscopic Examination of London Water. London: 1850.

AINSWORTH (William). Observations on the Pestilential Cholera (Cholera Asphyxia) as it appeared in Sunderland in the Months of November and December 1831.

Papers respecting the Origin, &c., of Yellow Fever in Bermuda in 1853. London: 1855.

BUDD (William, M.D.). Cholera: its Cause and Prevention. Association Medical Journal: 1854 and 1855.

CARPENTER (Alfred). Impure Water a Cause of Disease. Association Medical Journal, October 6, 1854.

LINDSAY (W. Lauder, M.D.). Experiments on the Communicability of Cholera to the Lower Animals. Edinburgh Med. and Surg. Journal: 1854.

MITCHELL (John). On the Falsifications of Foods: Article—Water and its Impurities.

Letter from the President of the General Board of Health to Viscount Palmerston, accompanying a Report from Dr. Sutherland on Epidemic Cholera in the Metropolis in 1854. Blue Book: 1855.

Report of the General Board of Health of the supply of Water to the Metropolis. Blue Book: 1850.

BLACKBURNE (William, M.D.). Scarlet Fever, and the Origin of Acute Contagions. London: 1803.

The Registrar-General's Weekly Returns for 1854: England.

SHAPTER (Thomas, M.D.). Sanitary Measures and their Results. London and Exeter: 1853.

Minutes of Information collected with reference to Works for the Removal of Soil, Water, or Drainage of Dwelling Houses and Public Edifices. Published by the Board of Heath: 1852.

BECKER (F. W., M.D., F.R.S.). Letters on Cholera in Prussia in 1831. London: 1832.

whom we refer, goes further, and adds to a statement of a presumed fact an explanatory theory. The cholera, according to him, like small-pox and cow-pox, is, under favourable conditions, a self-propagating disorder. The effects of the disease are due, he maintains, to a specific cell, which, introduced into the alimentary canal of the human body, is there rapidly reproduced, and withdraws fluid from the blood circulating in the capillaries of the intestines, by a power analogous to that by which the epithelial cells of the various organs abstract their secretions in the healthy body.

Thus reproduced, and thus extracting fluid in immense quantities from the capillaries, and drawing it into the alimentary canal, the cholera cell is soon abundantly thrown off in the free alvine discharges and vomitings, which so prominently characterise the choleraic disorder. But its effects may not rest here; the cholera cell, eliminated from one person, may be carried to some other person previously healthy; and, being drank, may excite the disease, as before, in a second victim; and so on and on, till there is developed an organised and general epidemic.

The above in a few words is the full meaning of the theory of cholera proposed by Dr. Snow; and this once admitted, the remainder of his explanation of the spread of cholera is easy. By a faulty system of house drainage, and by a neglect of cleanliness, the excreta of the cholera patient, charged with the germs of the disease, find their way on to the hands of dirty attendants, into drinking utensils, into cesspools, and into rivers, thence into water-pipes and houses, and so by some of these means into one human body, and then into another, but killing the former perchance as a step preparatory to doing the same for the latter.

Rather a disagreeable theory this, we believe, for sensitive old ladies during cholera epidemics. If cholera must come, it must; but surely not after this fashion. It is adding gall to vinegar. Any way else, king Cholera, enter into and upset the life balance of thy victims; but why choose this horrid and loathsome plan for attacking poor humanity?

We may then, for distinction's sake, call this supposed process the *objectionable* way of taking cholera; but if cholera likes to be thus doubly disagreeable, we cannot avoid it. The question is, is it so? And, after all, is Dr. Snow right?

Of course many objections have been and may still be raised against this theory; and to these we shall refer before alluding to any facts connected with the supply of water generally in its relations to disease.

First, it has been argued that the presumed cholera cell cannot be detected by the microscope, or by any chemical analysis. This argument is at once and to a certain extent satisfactorily met by the statement, that the virus of small-pox can only be recognised by its effects, and not by its physical properties.

Secondly, it is asked, Where does the first cholera cell come from? How many years have elapsed since the great ancestor of myriads of succeeding cells developed himself? Whence did he draw the elements of his existence? These questions, says Dr. Snow naïvely enough, "I will answer satisfactorily to any one who will first explain to me the origin of the first tiger or the first upas-tree." This is certainly no answer; but the conditions proposed are fair, and are not very likely to be accepted.

Thirdly, the question has been raised—Why, if the cholera cell is thus easily propagated and distributed, does it not always continue to indicate its presence like small-pox, which is more difficult of cultivation? The answer is, that neither a metropolis, nor a country, nor a continent, is capable of supplying cholera with victims, except for a time; nay, "the world is scarcely large enough for it; and were it not for its pasture in India, it would be in danger of passing out of existence, like the dodo of the Mauritius."

Fourthly, the leading question has been asked, Will the excreta of cholera produce cholera if taken into the stomach of healthy animals? Bearing on this subject we have but few facts—none that are affirmative, and some which are, if anything, negative. A few cases are recorded by Becker. On the 5th of September 1853, four young physicians tasted the intestinal fluid of a cholera patient. One of them, who had a diarrhœa previously, took cholera on the 8th, and died in the evening. A second was taken ill on the 9th with symptoms resembling cholera, and terminating in typhoid fever. M. Scipion Pinel also inoculated himself into a vein with the blood and intestinal contents of a cholera patient. Two hours afterwards he was sick and giddy, but recovered after free perspiration. M. Foy breathed the air expired by a cholera patient and tasted the vomited matters. He was unwell for four days, but did not take the disease. An assistant in the cholera hospital, (No. 2) Berlin, tasted the intestinal contents of cholera. Six hours afterwards he felt unusual motions of the bowels. After the eleventh hour diarrhœa came on, and three days later a smart attack of cholera. He had all through continued to perform his fatiguing duties in the hospital. Next, there is an instance referred to by Dr. Snow himself, and related by Messrs. Pearce and Marston of Newcastle, in which a dispenser at the Dispensary drank by mistake some rice-water evacuation without any effect whatever;—a result which Dr. Snow not very satisfactorily explains by saying that the large quantity of evacuation swallowed might have prevented its action. For, he argues, "it must be remembered that the effects of a morbid poison are never due to what first enters the system, but to the crop or progeny produced from this during a period of reproduction, termed the period of incubation; and if a whole sack of grain or seed were put into a hole in the ground, it is very doubtful whether any crop whatever would be produced."

Again, Dr. Thiersch of Munich has shown that cholera excreta, although not at first capable of producing cholera, did bring on symptoms of this disorder, when, after being kept from six to eight days, they were administered to some white mice.

Lastly, we have before us a most elaborate series of experiments in Dr. Lauder Lindsay's admirable essays on the communicability of cholera. In these experiments, Dr. Lindsay exposed several dogs and cats to all the supposed influences of cholera; these animals swallowed the excreta of cholera, and two or three died at particular periods with symptoms of cholera. At the same time, the majority recovered; and it is not quite clear that those which died did so simply from the effects of a specific cholera poison introduced into their stomachs.

These, then, are the experimental facts for and against the presence of a specific poison in the cholera excreta. We have only to remark that they require repetition, and to suggest that in future experiments animals, such as pigs, which are susceptible of small-pox and other zymotic diseases, and not cats and dogs, whose alimentary physiology is very different from that of man, should be the subjects operated upon.

Fifthly, an objection has been made to the theory we are now discussing, that if the specific cholera poison were really transmitted in the way described, every one who drinks the water impregnated with it ought to have the disease. These objections, Dr. Snow observes, "arise from mistaking the department of science to which the communication of cholera belongs; and from looking on it as a question of chemistry, instead of one of natural history, as it undoubtedly is. It cannot be supposed that a morbid poison, which has the property under suitable circumstances of reproducing its kind, should be capable of being diluted indefinitely in water, like a chemical salt, and therefore it is not to be presumed that the cholera poison would be equally diffused through every particle of the water." This is a reasonable defence, granting the general doctrine to be correct.

Sixthly, there are arguments in opposition to this hypothesis, based on the statements that cholera cases arise under circumstances which render it utterly impossible for the disease to have been communicated in the manner supposed, *i. e.*, isolated cases have occurred in which the patient could by no chance have swallowed the specific poison. Dr. Sutherland, in his elaborate report, concludes this part of his subject by the affirmation "that the evidence from the experience of the epidemics of 1849 and 1854 yields no sufficient proof that water is the specific cause of cholera."

But, passing from cholera to other diseases of the epidemic and endemic types, Dr. Snow is of opinion that some of these too, such as yellow fever, typhoid fever, and ague, are propagated by water. To this hypothesis also there have been raised objections similar to those named above.

Reviewing, without any bias for or against it, the theory of the specific nature of the cholera poison, as derived from the arguments already given, we may state that as a physiological speculation it is based on pretty correct views, and presupposes nothing that is at variance with the acknowledged laws of the animal economy.

It is now a well ascertained fact, that most agents which, when absorbed into the body, are capable of producing poisonous effects, may find an introduction as well by one portion of the mucous membrane as by another, as well by the stomach as by the lungs, and *vice versâ.* Nay, the skin itself, under certain conditions, will receive and transmit into the system various substances. If, therefore, there be a specific poison at all, either of cholera, yellow fever, ague, or other similar disease, there can be no doubt that such an agent may, if carried either into the lungs or stomach, enter into the organism; and by setting up a new series of molecular changes, induce its specific disorder. Viewed in its simplest sense, the fact is, that the mucous membranes throughout the body are all constituted to perform the same offices;—the functions of secretion and elimination; *i. e.*, they take in and give out. Thus prussic acid is absorbed into the body, and produces its peculiar effects, however it may be applied, whether to the mucous surface of the eyeball, to the stomach, or to the bronchial membrane.

With these facts before us, one inference may therefore be drawn, that Dr. Snow and his objectors are both right in the main; and that while the specific poison of cholera (for we must presume the existence of such a poison, though we may not understand its nature), may, by accident, be carried into the intestinal canal by the medium of water, it may also be wafted into the lungs by the medium of the air.

Many of Dr. Snow's objectors would, we doubt not, join us in this view of the case; which is more than we could expect from him, since it is opposed to his essential idea, that the specific cholera cell must be in contact with the intestinal surface, in order to produce the specific process of extracting water from the blood circulating in the capillaries; indeed, he lays this principle down as an axiom with regard to cholera, in the statement "that as cholera commences with an affection of the alimentary canal, and as the blood is not under the influence of any poison in the early stages of the disease, it follows that the morbid material producing cholera must be introduced into the alimentary canal."

Now, although, as it will be inferred, we are prepared to go great lengths with Dr. Snow in support of his peculiar views, we are obliged to stop whenever we meet with this absolute conclusion. Granting, for the sake of argument, that cholera, yellow fever, and ague, do arise from specific poisons; granting, too, that such poisons may act by being swallowed, we hesitate at present to accept the exclusive hypothesis that these poisons *must* be introduced into the alimentary canal to produce their peculiar effects. Profuse loss of fluid from the alimentary surface is the marked symptom of cholera; *ergo*, according to this author, the poison must be received by the alimentary canal. But if the cause of ague is often introduced into the system by the alimentary canal, then, by the same reasoning, ought the symptoms of ague to commence in this canal under such circumstances; which is not the fact. But one of the most marked symptoms of ague is profuse excretion from the skin, which excretion is, in fact, equivalent to excretion from the bowels in cholera ; *ergo*, according to the same author, the specific cause of ague should be a cell, which, being brought into contact with the external excreting surface of the body, should act on it as an "irritant," or, what is more probable, by withdrawing fluid from the blood circulating in the capillaries "by a power analogous to that by which the epithelial cells of the various organs abstract the different secretions of the body."

We presume no one will contend for this argument; and of course Dr. Snow would not, because he believes that ague, the marked sign of which is profuse evacuation from the skin, may be caused by the reception into the stomach of its specific cause. But, again, we know that many substances, which, when introduced into the alimentary canal produce free excretion, as the cholera poison may be supposed to do, bring about just the same result when introduced into the system by other channels ; thus elaterium introduced through the external surface of the body will lead to violent alvine action ; the inhalation of the animal effluvia from a dissecting room will cause the same result, and that to an extent sufficient to destroy life (as we have ourselves seen) ; and again, the inhalation of some narcotic vapours will induce vomiting and temporary purgation. We might push this argument much further, by showing that the specific action of many medicinal substances is brought about in an indirect manner, or by illustrating that some excretory organs, such as the kidney, can only be excited to over-secretion through the medium of some other organ and the blood.

The view that the specific cause of cholera may be carried by the air into the lungs, we are glad to see supported by Dr. Wm. Budd (who is a firm believer in the general truth of Dr. Snow's hypothesis). In his admirable and scholarly letters, Dr. Budd offers many remarkable facts in support of this argument.

The peculiarity and value of Dr. Snow's hypothesis as to the specific nature of the cholera poison, irrespective altogether of any particular mode by which the said poison is introduced into the system, has this advantage—that it supposes the existence of a certain cause ; and that it is supported by analogical reasoning derived from the observation of other spreading diseases, such as small-pox, the communicable nature of which is fully understood. In this regard it ranks higher than those speculations which assign, as causes of choleraic disorder, some unimaginable and perfectly unintelligible atmospheric influence, or the generation of some unknown and unappreciable agent in sewers and foul localities, which comes no one knows how, and goes no one knows whither, and, though generally diffused while present, attacks but a few individuals, comparatively speaking. True, the idea of a specific poison is as yet only hypothetical; still it is better, out of two, or rather out of two dozen hypotheses, to choose the simplest, and not let it escape until, by further observation, it is either wholly disproved or confirmed.

But, setting aside all further arguments of a theoretical nature, the question of water supply, in its bearings on disease, is one of great general importance. And in this sense, as we have said before, it is in no way a novel question.

Of the diseases which have been thought to arise from this source we may name malignant fever, typhoid fever, remittent fever, ague, plague, dysentery, cholera, the entozoa, glandular enlargement of the neck (goître, or full throat), paralysis, and yellow fever—diseases very different in character, and each springing from agents also of a different kind, some organic, others inorganic, but all conveyed by the watery medium, in which, according to their natures, they are either mechanically suspended or chemically dissolved.

Let us follow out this subject in reference to two or three of the diseases we have named.

*Goitre.*—With regard to glandular enlargements as resulting from the effects of impure water, here is a telling history condensed from the paper of Professor Johnston.

In the year 1843, a well was sunk in Durham Jail for the use of the prisoners. At the time no analysis was made; but during the succeeding ten years in which it was used, there arose from time to time certain peculiarities amongst the inmates of the prison; they equally suffered from glandular enlargements in their necks. After considerable time Mr. Shaw, the intelligent medical officer of the prison, began to suspect that the water drunken was intimately connected with the disorder. In July 1853, in consequence of the pumping machinery being out of order, the inmates were supplied from the river Wear. Immediately the affection of the neck began generally to abate, and ceased altogether to appear. An analysis of the water was now made, and it was found to contain not less than seventy-seven grains of solid matter to the imperial gallon, or from two to three times more than is usually considered proper for water in domestic use. The predominating ingredients of this solid matter were gypsum and magnesian salts.

Although the physiological and chemical sciences are not yet able to say whether the glandular enlargement in these cases was due to any *special* salt in this water, it illustrates the interesting point that a superabundance of solid matters in drinking-water may lead to the development of goître or full throat, a disease peculiar to some parts of Derbyshire and to the Swiss mountains, localities the waters of which are strongly impregnated with magnesian salts.

*Ague.*—As evidence in favour of the opinion that ague may result from the drinking of impure water, here is an interesting and striking example, recorded by M. Boudin, and related by Mr. Grainger.

In July 1834, eight hundred soldiers, all in good health, embarked on the same day in three transports at Bona, in Algeria, and arrived together at Marseilles. They were exposed to the same atmospheric influence, and were, with one essential difference, supplied with the same food and subject to the same discipline. On board one of the vessels were 120 soldiers; of these, 13 died on the passage from a destructive fever, and 98 more were taken to the military hospital of the lazaretto at Marseilles, presenting all the pathological characters proper to marshy localities. On seeing the physiognomy of these patients, altogether so unusual for Marseilles, one would have said that the Gulf of Mexico, the Delta of the Ganges, and the marshes of Senegal and of Holland, had supplied passengers to this ship. In short, by the side of a simple intermittent, there was a pernicious fever. On an inquiry being made, it was ascertained that on board the affected ship the water supplied for the soldiers, owing to the haste of embarkation, had been taken from a marshy place near Bona; whilst the crew, *not one of whom was attacked*, were supplied with wholesome water. It further appeared, that nine soldiers who escaped had purchased water of the crew, and had consequently not drunk the marshy water; and not a single soldier or sailor of the other two transports, who were supplied with pure water, suffered.

*Paralysis.*—Of the power of water in conveying the poisonous oxide of lead off leaden pipes and into the animal system, thus giving rise to paralysis and bowel disorder, there are many proofs. The danger of these pipes, which was known to Galen and Vitruvius, is one of no trifling moment. Dr. Christison relates, that some forty-five years ago a plumber undertook to supply the town of Tunbridge with water through leaden pipes, and laid down a course of pipes for a quarter of a mile. In the subsequent year an epidemic, of lead colic, occurred amongst the inhabitants, and one lady was paralysed in consequence for some months. Iron pipes were now substituted for the leaden, and the disease was permanently checked.

*Diarrhœa and Fever.*—As regards the effects of water supply in relation to diarrhœa and what is absurdly called continued fever, we have some striking facts. Here is one, related by Mr. Carpenter of Croydon:—In the year 1849, Croydon suffered severely from cholera. At this time the town was supplied with water from shallow wells and filthy ponds, the wells being merely drain shafts for the porous gravel upon which the town is built, and each generally in close proximity to a cesspool. In 1852 this system was altered; a proper water supply being derived from a well dug some fifty feet into the chalk. From this well a constant water supply is pumped into the town constantly, by a powerful steam engine. In the wet summer and autumn of 1852 the new water supply was interfered with, and the people became greatly prejudiced against it. The old wells were now filled with water strongly impregnated with animal matters of the most odious description, derived from the cesspools. An outcry was raised against the new supply, and great numbers of persons flew to those very wells which contained so large an amount of decomposing animal substance. At this moment a frightful epidemic of typhoid fever appeared, which was falsely attributed to the new pipe sewerage laid down by the Board of Health. In the

Park-lane of this town there is a large school belonging to the Society of Friends. This school is kept scrupulously clean, and free from nuisances of every kind : ventilation, drainage, and everything connected with sanitary arrangements seemed excellent; yet the scholars suffered very much from the epidemic. Eventually the school was dismissed. On its reassembling in the spring of 1853, the health of the children continued pretty good; occasional slight cases of illness occurred, but not more than the average would be among so many. In March 1854, diarrhœa appeared, slight at first, but in a few days rapidly increased. The patients all complained alike, and peculiar feverish symptoms also presented themselves. On inquiry into the cause of these symptoms, Mr. Carpenter and his partner, Mr. Westall, found that the water drunken was derived not from the mains of the water works but from a well. The water was therefore changed. On the day on which the change was made there were sixteen fresh cases; on the following day there were two only; and eighteen hours after the change had been effected not a single fresh case came under notice. In the course of a few days the majority had recovered. On a minute examination of the sewerage, one of the drains passing from a wing of the building and some of the out-houses was found blocked up, and extensive leakage had taken place into the ground around the impervious sewer, which, unfortunately, ran in close proximity to the well, a pervious gravelly soil only intervening.

*Yellow Fever.*—On the subject of yellow fever and water supply but little can be said of a positive kind. Certain it is, however, that wherever yellow fever has appeared, there has the water been in a filthy and faulty state.

There lies before us at this moment, one of the latest reports on this disease, entitled *Papers respecting the origin of Yellow Fever in Bermuda in* 1853. By the most admirable example of placing the "wrong men in the wrong place," the drawing up of this medical report was confided to—to whom does the reader think ? to a lieutenant-colonel and two lawyers ! members of professions which, of all others, least represent competency for such a task. However, this report, as it contains the evidence of nearly sixty witnesses of various kinds, mayors, barrack-masters, vestry men, clergymen, private soldiers, magistrates, pilots, and doctors, has in it, with all its omissions and necessarily scientific defects, many important facts, none of which are more interesting than some which incidentally (and without the slightest reference to water as even a possible cause of the epidemic) relate to the water supply of those localities in which the epidemic raged most fiercely.

In these incidental remarks we find abundant evidence of an intimate connexion between bad water and the virulence of the disease. Mr. Innes, in remarking on the disease in Fort Cunningham, Bermuda, tells us of some tank water which supplied one end of the barracks, and which at last became so offensive that it could not be used. The great mortality which took place in this quarter of the barracks he attributes to the effluvia these tanks.

Further on we gather from a sanitary report drawn up for the commissioners by the naval store-keeper, the deputy-inspector of hospitals and the clerk of the works, as well as from the evidence of John D. Anderson, Esq., C.E., that during the prevalence of the epidemic in the localities of Ireland Island and the towns of Hamilton and St. George, Bermuda, there was no proper arrangement for the supply of water at all, and that such water as was used was of necessity impure. There were three or four wells i[illegible] Hamilton, says Mr. Anderson, but the water was only used by [illegible] poorest classes, its quality being indifferent. In the majorit[illegible] cases the water supply was derived from tanks which received [illegible] rain or dew after it had passed over shingle-roofs erected for c[illegible]ing it. Many of these tanks were placed under the lower flo[illegible] houses, and some of them close to privies, from which they d[illegible] most loathsome impurities. The water of the tanks was gene[illegible] execrable, giving off sulphuretted hydrogen in large quantities, [illegible] that in some instances it could not be used for drinking, and had [illegible] be obtained from the wells. When to these facts is added the statements, that in Bermuda drainage and sewerage are neglected altogether; that the contents of privies and cesspools are allowed to accumulate in natural pits or hollows and on the ground surface, whence they are only removed by the washing of the sea; and that the tanks are filled only from water collected on roofs, or from percolation through an impure soil, there is not wanted, we imagine, much further evidence to prove that the effects of the vile water, which must at the time of the pestilence have been hourly consumed, had no small share in intensifying, if not producing, the disease. Yet the commissioners have entirely omitted the investigation of this question; and one of them has profoundly explained his view of the cause of the disease, by comparing it to a poison cloud, which, passing from city to city, and country to country, leaves and scatters poison germs, for the development of which "localising" conditions afford the necessary nidus.

*Cholera.*—But if it is evident that the causes of the above named diseases may be propagated by impure water, the proofs of the cholera poison being carried by this medium are still more obvious. On this point, most observers agree to a certain extent. Thus Dr. Greenhow, who has urged stronger reasons than any one else against the idea of the specific poison of cholera being conveyed by water, states in Dr. Sutherland's report, "that although unwholesome water very much aggravates the result, it is only one cause amongst several." And Dr. Sutherland, who obviously treats Dr. Snow's labours slightingly, since he refers to that gentleman's theory without even mentioning his name, sums up his report by saying that the use of water containing organic matter in a state of decomposition is one predisposing cause of cholera, and that the use of such water has aggravated the severity of the late epidemic, especially in the districts south of the Thames.

In another place, too, Dr. Sutherland records an instance which occurred under his own observation. A street in Salford contained ninety houses. The inhabitants of thirty of these houses used water from a well into which a sewer had leaked, and among them there were nineteen cases of diarrhœa, twenty-six cases of cholera, and twenty-five deaths; while in the remaining sixty houses, which derived their water supply from purer sources, there were eleven cases of diarrhœa, but neither cholera cases nor deaths.

But the following is the grand argument of Dr. Snow, as to the [illegible]ects of water supply on the progress of cholera in London in 1854. [illegible]here is a portion of the great metropolis which takes in sixteen [illegible] districts, called respectively Christchurch, Southwark; Kent [illegible]; Borough Road; London Road; Trinity, Newington; St. [illegible]'s, Walworth; St. Mary, Newington; Waterloo Road, 1st [illegible] Waterloo Road, 2nd part; Lambeth Church, 1st part; [illegible]beth Church, 2nd part; Kennington, 1st part and 2nd parts; [illegible]xton; Clapham; and St. George, Camberwell. In the district [illegible]cluding these sub-districts, there resided in 1854 upwards of [illegible]00,000 people, of various ages and occupations. This population received its water supply from two sources; from the Southwark and Vauxhall Company, which collects its supply at Battersea Bridge, and contains immense quantities of the sewage of London; and from the Lambeth Company, which obtains its supply from Thames Ditton, a pure source. Here, then, was an experiment based on a grand scale; cholera was present, and three hundred thousand persons were as regards water supply divided into two groups; one group drinking water containing the sewage of London, the other having water quite free of such impurity.

Let us now see what was the result. If the facts collected with great labour by Dr. Snow are to be trusted as we now have them before us, they state in simple terms that in the first four weeks of the epidemic the proportion of fatal attacks to each 10,000 persons supplied by these different waters was as follows: Southwark and Vauxhall, 71; Lambeth, 5. In other words, the cholera was fourteen times as fatal at this period amongst persons having impure water, as amongst other persons subject to the same local influences, except that of water supply.

We forbear from giving less significant examples than the above of the influence of water in regard to cholera, and we are willing to accept even the one we have selected, with all reasonable modifications and subtractions. But, apart from all theory, should time prove the statement to be mainly correct, it must be set down as one of the most important demonstrations ever brought forward of the effects of an impure water on a vast population.

But here our task, for the present, at all events, must end. We have wished only to lay the great facts of the influence of water on health plainly before the reader; leaving him to apply the knowledge which the narration of such facts affords to some practical purpose in relation to sanitary improvements.

*B. W. Richardson.*

